# Assessing the Need for Semantic Data Integration for Surgical Biobanks—A Knowledge Representation Perspective

**DOI:** 10.3390/jpm12050757

**Published:** 2022-05-07

**Authors:** Mathias Brochhausen, Justin M. Whorton, Cilia E. Zayas, Monica P. Kimbrell, Sarah J. Bost, Nitya Singh, Christoph Brochhausen, Kevin W. Sexton, Bernd Blobel

**Affiliations:** 1 Department of Biomedical Informatics, University of Arkansas for Medical Sciences, Little Rock, AR 72205, USA; jmwhorton@uams.edu (J.M.W.); czayas@uams.edu (C.E.Z.); kevin.sexton@uams.edu (K.W.S.); 2 Trauma Performance Improvement Coordinator, University of Arkansas for Medical Sciences, Little Rock, AR 72205, USA; mpkimbrell@uams.edu; 3 Department of Health Outcomes and Biomedical Informatics, University of Florida, Gainesville, FL 32611, USA; sarah.bost@ufl.edu; 4 Department of Animal Sciences, University of Florida, Gainesville, FL 32611, USA; nitya11@epi.ufl.edu; 5 Emerging Pathogens Institute, University of Florida, Gainesville, FL 32611, USA; 6 Central Biobank, Institute of Pathology, University and University Clinic of Regensburg, 93053 Regensburg, Germany; christoph.brochhausen@klinik.uni-regensburg.de; 7 Department of Surgery, University of Arkansas for Medical Sciences, Little Rock, AR 72205, USA; 8 Institute for Digital Health & Innovation, University of Arkansas for Medical Sciences, Little Rock, AR 72205, USA; 9 BioVentures LLC, Little Rock, AR 72205, USA; 10 Medical Faculty, University of Regensburg, 93053 Regensburg, Germany; bernd.blobel@klinik.uni-regensburg.de; 11 eHealth Competence Center Bavaria, Deggendorf Institute of Technology, 94469 Deggendorf, Germany; 12 First Medical Faculty, Charles University, 11636 Prague 1, Czech Republic

**Keywords:** surgical biobank, post-traumatic arthritis, osteomyelitis, semantic data integration, system theory, biomedical ontologies, knowledge representation

## Abstract

To improve patient outcomes after trauma, the need to decrypt the post-traumatic immune response has been identified. One prerequisite to drive advancement in understanding that domain is the implementation of surgical biobanks. This paper focuses on the outcomes of patients with one of two diagnoses: post-traumatic arthritis and osteomyelitis. In creating surgical biobanks, currently, many obstacles must be overcome. Roadblocks exist around scoping of data that is to be collected, and the semantic integration of these data. In this paper, the generic component model and the Semantic Web technology stack are used to solve issues related to data integration. The results are twofold: (a) a scoping analysis of data and the ontologies required to harmonize and integrate it, and (b) resolution of common data integration issues in integrating data relevant to trauma surgery.

## 1. Introduction

Trauma is the leading cause of death and disability for patients less than 45 years old [1]. Recently, the need to decrypt the post-traumatic immune response to improve patient outcomes has been identified [2,3,4,5]. One strategy proposed to achieve this is large fluidics biobanks [6]. Along with other researchers [7], we advocate for more expansive surgical biobanks, including tissue. One of the core issues when building a trauma-oriented surgical biobank is integrating patient data from other healthcare providers. This is a generic data management problem regarding biobanking [8].

Implementation, maintenance, and use of a surgical biobank are mandatory, among other things, for a better understanding of the post-traumatic immune response, for instance, in patients with multiple injuries. We aim at improving secondary data analysis to assess factors contributing to complications. Since patients with numerous injuries are frequently transferred from lower acuity facilities to a more specialized or higher-level trauma center, the data management activities of a surgical biobank need to include collecting and harmonizing data from all trauma care providers of the specimen donors. Collecting data from lower acuity facilities to trauma centers is crucial to identify potentially specific pathophysiological or new prognostic factors.

In this paper, we aim to achieve two goals:

Propose system architecture considerations for a surgical biobank against the background of crucial data needs to address post-surgical arthritis and osteomyelitis.

Demonstrate how semantic analysis guided by the generic component model (GCM) informs harmonization of clinical and clinically relevant data regarding post-traumatic arthritis and osteomyelitis in a surgical biobank. Thereby, we highlight the importance of definition, representation, and integration of the underlying concept spaces (ontologies) of the different domains involved, from which the data can be derived, represented, and implemented, but also the current context [9].

In the first step of our analysis, we will assess the patterns of complex collaborations in trauma patient triage and care using a systems theory approach. This approach will assist us in determining the scope of data collection and data harmonization across multiple healthcare providers. This research can aid in the development of IT infrastructure to support the implementation, maintenance, and management of a surgical biobank. To provide actionable information, we will suggest current domain ontologies that are applicable to our use cases.

In the second step, we will illustrate how Semantic Web technologies can resolve common data collection challenges related to surgical trauma: (a) issues created by restrictions specified in local information models, (b) issues created by the need to integrate data from heterogeneous sources, and c) issues created in the process of data entry.

## 2. Data Needs of Surgical Biobanks

Our research focuses on musculoskeletal injuries, one of the most common injury patterns leading to disability after trauma. These injuries are almost exclusively repaired surgically, making this the ideal population for creating a surgical biobank. As part of the fracture repair process, we can easily collect samples of bone, skin, subcutaneous tissue, small veins, and muscle without impact on the patients’ recovery. Additionally, joint replacement is a common procedure allowing for age-matched control sample collection of similar tissues. Interestingly, the main indication for joint replacement is arthritis, a known complication of orthopedic trauma, thus allowing us to collect samples across the spectrum of life and investigate the mechanisms of post-traumatic arthritis. This is not our only outcome of interest, however, as infection, such as osteomyelitis or soft tissue infection, is the most common early complication of fracture repair. Diagnosis of both post-traumatic arthritis or osteomyelitis occurs after initial treatment for a fracture, and the reasons for the occurrences are not completely understood. Due to this, treatment for these diagnoses frequently occurs at different trauma care facilities from the initial treatment. In this context, it is desirable to establish predictive and prognostic factors for future therapeutic strategies to avoid numerous repetitive surgical interventions or amputations in complicated trauma patients. For this purpose, surgical biobanks collecting relevant biomaterials and comprehensive clinical and laboratory data are needed. The complete monitoring of laboratory, clinical, and imaging data as well as the historical information of primary care, transportation times, and duration of treatments are relevant. To reach this goal, data from different sources should be brought together with the biospecimens to curate data-rich biobank specimens.

The lack of a unified coding schema and of structured data for information relevant to address the pathogenesis of individual cases creates additional difficulty for data harmonization in this domain. While there are emerging classification systems, no widely accepted standard exists. In all likelihood, any standards set quickly, would continue to change and evolve as our understanding of precision medicine in trauma increases. Thus, the problem of lack of comparable data for mid- and long-term studies would be proliferated for years to come. Since the existence of a well-developed understanding is a requirement for creating stable standards, it is obvious that we cannot use standards to collect, curate, and maintain the data we are collecting with the goal of developing our understanding of precision medicine in trauma. Challenges in harmonizing data are frequently found regarding treatment and procedure information, demographic information, problem lists, medication lists, and organizational structures of trauma centers [10,11,12] or healthcare providers involved in the initial treatment.

## 3. Integration Challenges Created by Restrictions of Local Information Models

A common practice is that each trauma patient is admitted to the nearest hospital that participates in the regional or state trauma program. However, complications such as infections and prolonged or failed bone or wound healing due to complicated trauma or to known, or even unrecognized, primary diseases, such as diabetes, hypertension, or arteriosclerosis, make the transfer to a more specialized or higher-level trauma center necessary [13]. In these centers, an interdisciplinary team will review previous laboratory as well as clinical tests. Recent and earlier imaging data will be compared, and commonly, a surgical revision is necessary. With respect to the management of specimens acquired during that healthcare process, one data integration problem frequently occurs: The entry number of a specimen is commonly used as the reference number. Over the course of treatment, especially with complications, a patient may have several entries, each receiving a new entry number. These samples are identified and documented in the so-called sample history as follow-up samples for the initial injury. Typically, the sample history assignment is performed using the patient identifier. Many systems enforce that there be only one patient identifier for each patient. If the patient has all samples taken at the same healthcare institution, this does not present a problem. However, if some of the samplings are performed at another healthcare provider, tracing disease progression via the patient identifier is no longer possible because the entry number cannot be matched with the patient identifier at the location of the surgical biobank.

## 4. Integration Challenges Created by the Need to Integrate Data from Heterogeneous Sources

The University of Arkansas for Medical Sciences (UAMS) Medical Center is the only adult Level 1 Trauma Center with verification by the American College of Surgeons in Arkansas and sees over 3000 trauma-related patients annually, with admission exceeding 2000 trauma-related admissions. The medical center is the only tertiary care center in the state and has 24 trauma and surgical intensive care unit beds. In collaboration with the surgical services, the surgical critical care teams manage critically ill patients yearly across trauma surgery, burn surgery, emergency general surgery, surgical oncology, pancreas-biliary surgery, bariatric surgery, neurosurgery, orthopedics, obstetrics and gynecology, vascular surgery, otolaryngology, urology, plastic surgery, and transplantation surgery. Being the only Level 1 trauma center in the state of Arkansas, UAMS is an excellent example of a trauma care facility, receiving 560 orthopedic trauma patients annually. Additionally, they are a referral center for orthopedic patients with complications, including post-traumatic arthritis and osteomyelitis. The UAMS Trauma Database strives to collect, maintain, and store clinical data on patients in the acute care phase as well as readmission of their population within 30 days of discharge. For 2021, we had 18 readmissions within 30 days in our ortho population. This excludes patients with acute care at another hospital, who are subsequently referred for post-acute complications. Acute-phase trauma information on this selected population of Arkansas trauma patients with complications is limited to what is in the transferring records or the patient’s history and physical notes. These patients are not included in the UAMS Trauma Database, thus restricting orthopedic studies to those with initial acute trauma care at UAMS.

The Arkansas Trauma Registry (ATR) might be a potential source for tying all Arkansas trauma cases together. Challenges with using data from the ATR include variability in the collection of National Trauma Data Bank (NTDB) data elements and access to the data. In Arkansas, only Level 1 and Level 2 trauma center personnel receive annual training as part of the Trauma Quality Improvement Program (TQIP) [14]. Level 3 and Level 4 programs are encouraged to use the NTDB data dictionary for applicable NTDB data elements, but training is limited to a review of data dictionary changes at the Annual ATR Conference.

Even with TQIP training, variability of data collection is suspected to be present among Level 1 and Level 2 facilities due to different interpretation of definitions. To mitigate variability, the National Trauma Data Bank (NTDB) Data Dictionary [15] work group vets the dataset annually. This process includes refinement of data element terminology, plus revision and/or additions to definitions. Additionally, TQIP provides annual education update through their annual conference, and via web training modules/quizzes. This is why the educational opportunities offered by TQIP annually are relevant to data quality. 

Even with this, there remains some variation in data elements such as “unplanned OR”, which has been refined from “unplanned return to the OR” to “unplanned visit to the OR” (OR stands for operation room). Another refinement includes adding exclusions to the definition. The current definition is as follows:

“Patients with an unplanned operative procedure OR patients returned to the operating room after initial operative management of a related previous procedure” [15].

This definition leaves much open to interpretation. Some users interpret this as a surgery that is unexpected or due to some untoward event and not potentially expected events such as visits to the OR due to a failed limb salvage and similar clinical situations. This is an example of a clinical situation where the possibility that the patient may require surgery is inherited. There is anecdotal evidence that there is a tendency to subsume limb salvage failure and similar examples under the unplanned OR category. This highlights the potential for different interpretations and the lack of shared understanding of the data elements in the NTDS Data Dictionary.

## 5. Integration Challenges Created during Data Entry

Besides the accurate resolution of identifiers and the ability to semantically integrate data, information about the organizational structure of trauma care providers give important insight into potential effects on patient outcomes. Curating data about organizational structures in trauma care, in particular with controlled vocabularies and ontologies, is a relatively new area of research and development. The NIH-funded CAFÉ (Comparative Assessment Framework for Environments of Trauma Care (R01GM111324)) provides a controlled vocabulary to describe and assess organizational structures of trauma care providers [11]. In addition, the project created a web-based architecture to collect, manage, and store semantically rich information about trauma care organizations via a user-friendly online questionnaire with the Ontology of Organizational Structures of Trauma centers and Trauma systems (OOSTT) automatically representing the answers in a computer-interpretable language [10,12].

For validation purposes, the CAFÉ project invited stakeholders to enter data about trauma centers and trauma systems. In the first round of this activity, we collected data on two Level 1 trauma centers and one Level 2 trauma center. Table 1 shows the extracted results for two CAFÉ questions.

The answers from trauma centers A and C looked unlikely based on the CAFÉ team’s experience. Our interpretation of the situation is that the persons who entered the data for those two questions for those two centers were not certain of the difference between these two categories or they did not have access to data that provided a differentiation between board-eligible and board-certified emergency physicians. While OOSTT has a representation of both “board-certified emergency physician role” and “board-eligible emergency physician role”, only the first is formally fully defined, so that at the current state of semantic representation, a disambiguation was not possible. One factor contributing to the lack of clarity is that all board-certified emergency physicians were at some point (and remain to be) board-eligible. However, the intention behind this question is to find out how many emergency physicians are board-eligible, but have not yet been board-certified, as opposed to those who are already board-certified.

## 6. Methods

### 6.1. System Architecture Methodology: The Generic Component Model

**Figure 1:**
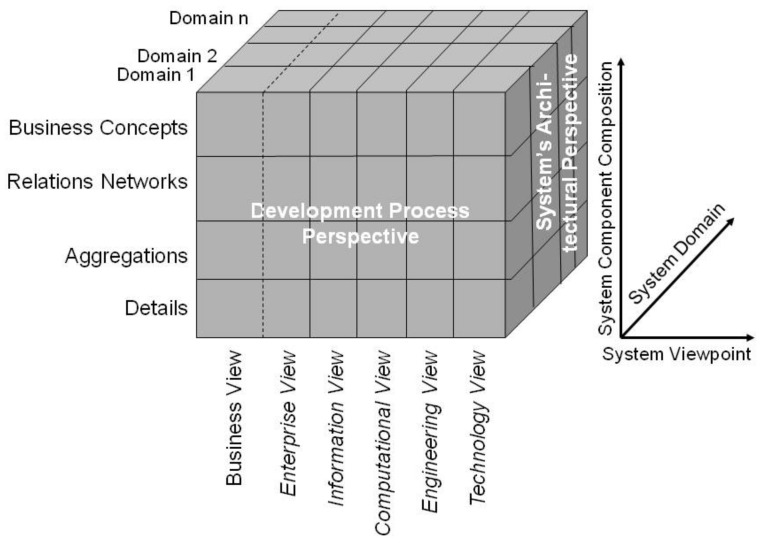
The generic component model. From: [18].

The GCM is a top-level architectural model for any multi-domain system, formally representing the system’s components, their functions, and interrelations structurally and behaviorally by a cube with three dimensions: (a) specific aspects/perspective (domains) of/on the system forming domain-specific sub-systems; (b) generic granularity levels of the system’s elements enabling the composition/decomposition of the system; (c) the viewpoints within the system’s development process (Figure 1). It is described and specified in ISO 23903 Interoperability and integration reference architecture—Model and framework [16,17]. For each business case, the subsystem components and their functions and interrelations are instantiated by naming and representing them using the specific terminologies and ontologies of the domains involved in that business case. For this reason, the GCM specifies a business view in addition to the five views defined by the ISO 10746 Open distributed processing—Reference model (RM-ODP) as starting point for a system development process [17]. The views prescribed by the RM-ODP are enterprise view (purpose, scope, and policies of the system), information view (information processing, semantics of information), computational view (functionality of the system, functional decomposition), engineering view (implementation, distribution of processing performed by the system), and technology view (choice of technology for the system), all represented using information and communication technology (ICT) ontologies [17,18]. Furthermore, ISO 23903 [16] introduces generic granularity levels for correctly representing and interrelating compositions/decompositions of elements, that way enabling integration of, and interoperability between, elements of different complexity. As only elements at the same granularity level can be interrelated, elements at different granularity level first have to be harmonized by composing or decomposing them. By adding the business view and granularity levels, the GCM enables the correct representation and management of multi-domain real-world systems, including supporting ICT solutions.

Applying the methods to the use case at hand first requires considering the role of the information models and the database schemata according to the views of the GCM. Both belong to the information view since their primary focus is to guide information processing within the system. Hence, they do not provide real-world knowledge, such as the fact that one person can have more than one patient ID and that sampling of tumor progression is frequently performed by different healthcare providers. More generally, due to the limitations of IT grammars, the correctness of representations of, and relations between, elements cannot be justified within this viewpoint. Notably, those aspects lie outside each electronic health record (EHR) system and are ill-fitted to be represented within the information view. However, from our database example, not accounting for those aspects of the business view may lead to errors in the system.

### 6.2. Semantic Integration Methods

Our challenge is to provide a semantically rich representation of clinical and clinically relevant data for a surgical biobank. This approach has been shown to be useful for integrating heterogeneous clinical data in an international cancer imaging repository, The Cancer Imaging Archive [19,20,21]. To create a semantically rich representation for biomedical data, we follow the recommendations outlined by Brochhausen et al. [22] The basic strategy is to use the Semantic Web stack [23] along with realism-based ontology development [24] as the general methodology to inform knowledge representation. Their approach is based on experiences with multiple implementations in biomedical informatics, which have influenced the evolution of their methodology from the beginning [25,26,27,28].

In our project, we are transforming clinical and clinically relevant data into the resource description framework (RDF) language before we load it into our RDF-based data management system of the biobank. RDF [29] is a Semantic Web standard that allows the representation of information in a machine-interpretable way. For each entity, RDF provides a unique identifier. RDF data can be annotated and used along with domain descriptions provided in the Web Ontology Language (OWL) [30]. We are using RDF representations and OWL ontologies following the methodology described by Smith and Ceusters [24] and the best-practice principles of the OBO Foundry [31,32]. 

In addition, we will use the GCM (Section 6.1) to assist knowledge representation. The GCM proves to be a fitting tool to help with selecting optimal knowledge representation for incoming data and guide the process of filling gaps in existing ontologies, based on the medical information models used to curate the data at the initial healthcare providers. While IT-oriented information models fulfil a crucial role in planning, defining, and describing the operational behavior of an IT system, such as an EHR system or a biobank information system, there is a lack of semantic capabilities of these models [21]. Due to the high level of abstraction and expressivity of information models, so rarely being complete and decidable, the resulting representation, especially regarding relationships, is frequently focused on data in that system alone and not on what the data represents, in our case the medical world, e.g., the patients, encounters, and prescriptions described in an EHR or the specimens, donors, and storage properties in a biobank, thereby including context and common-sense knowledge. Building an ontology or selecting from existing ontologies can lead to a mismatch between the system data model and the more holistic ontologies. In our approach, we use the GCM to overcome the implementation gap between healthcare-provider-specific information models and preexisting domain ontologies needed for the semantically rich representation of surgical biobank data.

In this paper, we follow the approach of Uribe et al. [33] used to represent a GCM-based generic model of a Type 2 diabetes mellitus care system. In parallel to their approach, we analyze the following three domains of the system: medical domain, policy domain, and resource domain.

- *Resource Domain—*represents all agents (humans, organizations, devices), means, and equipment to carry out activities (in our example, trauma care activities) in the system [33].
- *Policy Domain—*represents rules, regulations, and guidelines relevant to the system [33] (e.g., clinical guidelines, trauma system regulations, EHR regulations).
- *Medical Domain—*represents the medical and biological entities relevant to the system [33] (anatomical entities, treatments, diagnoses, etc.).

In this paper, we are not applying the GCM for a software development and implementation process, but restrict our considerations to the business view, following the approach by Uribe et al. [33].

## 7. Results

### 7.1. System Architecture Results

Using the GCM and applying it to analyze a medical care system for orthopedic trauma from the business view yields several interesting results. The findings we describe can help to inform the development of better communication and data management methods to close communication gaps that currently exist, particularly when it comes to the need for additional therapy, for osteomyelitis and post-surgical arthritis. Regarding our goal of establishing a surgical biobank to fill knowledge gaps regarding these two diagnoses, this analysis provides a survey regarding the relevant agents (healthcare providers), facilities, and guidelines. This enables a comprehensive assessment of the data needs of such a biobank.

**Figure 2:**
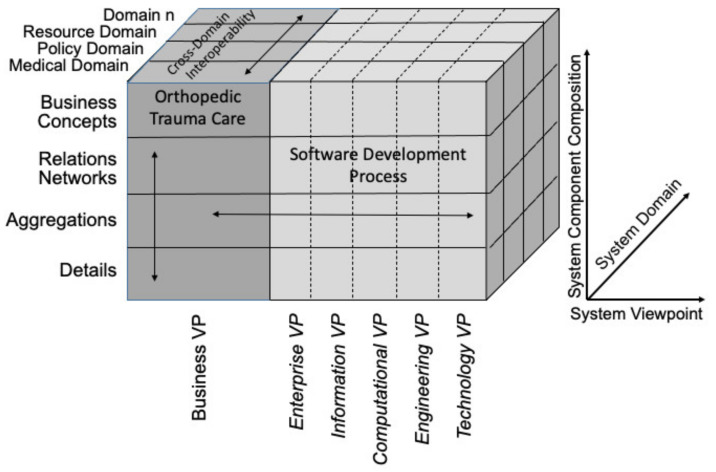
The GCM applied to analyze the business VP of orthopedic trauma care, following the approach used by Uribe et al. [33] for diabetes mellitus. Adapted from [33].

Figure 2 shows the domains relevant to orthopedic trauma care, in accordance with the GCM. Table 2 provides a list of examples of entities relevant to the GCM business view of orthopedic trauma care. These entities are sorted into the resource domain, policy domain, and medical domain. In addition, we provide a list of OBO Foundry [31,32] ontologies that provide a semantically rich representation of entities in those domains. This analysis allows for correct modeling of the inter-domain relationships to ensure cross-domain interoperability.

### 7.2. Semantic Integration Results

#### 7.2.1. Overcoming Issues Created by Local Information Models—Identifiers

Applying the methods to the use case of integration issues created by restrictions on identifiers in local information models requires consideration of these information models from the perspective of the GCM (see Section 6.1).

**Figure 3:**
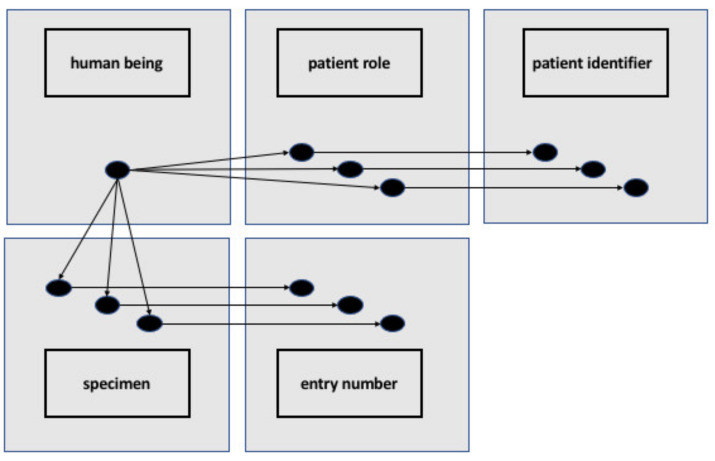
Representation of RDF individuals and OWL classes representing a human being having multiple patient roles, corresponding to multiple patient IDs and multiple specimens derived from that human being corresponding to multiple entry numbers. From: [39].

Figure 3 shows an RDF representation of specimen donor, specimen, patient role, and the relevant identifiers. There is no restriction on the numbers of identifiers, and the graph representation allows one to trace each identifier to what it identifies, and each patient’s role and each specimen to the person who is the specimen donor. The different classes and instances in our solution are modeled based on the best practice of realism-based ontology as described in Smith and Ceusters [24].

#### 7.2.2. Integration of Heterogeneous Data Sources

This problem represents the classical use case for Standard Widget Toolkits (SWTs) using the native graphical elements of the computer’s operating system: existing data models, common data elements, or dictionaries are heterogeneous or ambiguous. Solving this issue using SWTs rests on the idea of using computer systems to help disambiguate the data from heterogeneous sources. Bona et al. pointed out that this rests on an agreed understanding of what it means for a computer to *understand* the content [22]. Ultimately, the level of understanding proposed by Bona et al. is the ability to sort data points into data elements using SWTs [22].

The solution we propose here is to start with providing an RDF-style representation of “unplanned visit to the OR” that formally defines whether or not ER visits such as OR due to a failed limb salvage are an instance of an unplanned visit to the OR, or similar surgical procedures, or not. The disagreement is whether or not OR visits that might not have been initially planned, but that were always a final option, belong in this category. We propose to create a new OWL class for “surgical encounter” as a subclass of “health care encounter” (OGMS_0000097) from the Ontology for General Medical Science (OGMS) [40]. For the “surgical encounter” class, we propose the following textual definition: A healthcare encounter, where the goal is to provide a patient with surgical treatment.

In addition to expanding a class from OGMS, we also propose to expand the class “plan specification” (IAO_0000104) from the Information Artifact Ontology (IAO) [41] by creating a subclass “treatment plan specification”. For this class we propose the following textual definition: A plan specification that defines the objectives, actions, and healthcare encounters to treat the medical condition of one patient.

Now we can create a subclass of “surgical encounter” named “unplanned surgical encounter”, which we plan to represent “unplanned visit to the OR”, excluding visits such as OR due to a failed limb salvage. For this new class, we propose the following definition: A surgical encounter that does not realize the concretization of a plan specification which is part of a treatment plan.

We propose the following equivalence axiom for “unplanned surgical encounter”:

“surgical encounter”

and not realizes some (‘realizable entity’ and concretizes some (‘plan specification’ and ‘part of’ some ‘treatment plan’)) 

This axiom would ensure that any surgical procedures that are performed during healthcare encounters without being part of a treatment plan are not pulled into the class “unplanned surgical encounter”. In the example of failed limb salvage, the amputation of the limb under treatment is already a medical option in the treatment, although certainly not the most desirable outcome. Hence, any case of failed limb salvage would not be subsumed under an unplanned surgical encounter. This would disambiguate the current situation and resolve the lack of clarity, using a computational method.

#### 7.2.3. Resolving Problem Integration Issues Created during Data Entry

The solution to the lack of clarity between board-eligible emergency physicians and board-certified physicians we detected during the data entry for the CAFÉ project begins with OOSTT. The knowledge representation requirements of the CAFÉ project did not force the previous development of a class representing the individual emergency physicians, but the following classes were sufficient: “board-eligible emergency physician role” (OOSTT_00000173) and “board-certified emergency physician role” (OOSTT_00000171). To resolve the issue surrounding potential ambiguities during data entry, we created two novel classes in a new branch of the OOSTT ontology: “board-certified emergency physician” (OOSTT_00010000) and “board-eligible emergency physician” (OOSTT_00010001). These two classes have the following equivalence axioms:

“board certified emergency physician” (OOSTT_00010000):

 “Homo sapiens”

 and ‘bearer of” some “board-certified emergency physician role”

“board-eligible emergency physician’ (OOSTT_00010001):

 “Homo sapiens”

and (is_specified_output_of some “emergency medicine residency program”)

and (inverse (“is about”) some ‘compliance with state licensure requirement information content entity’).

**Figure 4:**
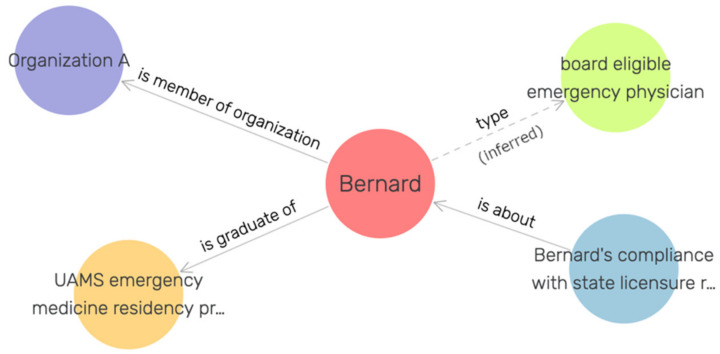
Example individual representing a human being, Bernard, who is board-eligible, but not yet board-certified.

After making these changes to the class structure of OOSTT, we created 100 test individuals to represent emergency physicians in 28 virtual trauma centers, some being board-eligible, some being board-certified. Figure 4 shows an example of the RDF representation of a board-eligible emergency physician. We loaded the resulting ontology and the individuals in a GraphDB triple store using ROBOT [42] to materialize class assertions. We queried this triple store with the following SPARQL queries to query out the board-certified individuals vs. the board-eligible individuals that are not (yet) board-certified.

## 8. Discussion

In applying a system architecture approach to orthopedic trauma care using the GCM, we follow Uribe et al. [33] to order the involved domains in the following sequence: medical, policy, and resource (Figure 2). This allows us to analyze and to represent the relationships between orthopedic trauma care (e.g., procedures) and the policy domain (e.g., clinical guidelines, state regulations). In placing the resource domain behind the policy domain, the focus of our analysis demonstrates how the resources (e.g., trauma centers, trauma personnel) relate to the policies, meaning which actions, requirements, and credentials the policies specify for trauma care resources. This order of the domain prioritizes the role of policy in the trauma care process, and the roles of the resources in those policies for our initial approach. This order is consistent with the accreditation practices of American College of Surgeons and state trauma systems. It is important to note that the ordering of the domains when analyzing a system is arbitrary and can be chosen according to the focus of the study. To analyze and represent how the trauma care personnel relates to the procedures, we will reorder our domains to focus on the cross-domain interoperability between the resource domain and the medical domain.

To resolve problems of data integration due to restrictions in local information models, we have applied both the GCM and methods of a realism-based ontology development. Notably, the successful deployment of ISO 23903 [17] for integrating different domains and knowledge spaces, including their specific models, has been demonstrated for the integration of HL7 privacy and security specifications [43] in ISO 13606 EHR communication [44], the harmonization of concepts from ISO 12967 [45] and ISO 13940 [46], or the mapping of open EHR (ISO 13606) archetypes [47], ISO 13972 clinical models [48], and HL7 FHIR resources [49].

The limitations of this study and the three use cases demonstrated lie in the fact that the work presented addresses issues from a knowledge representation perspective. The solutions presented are readily available to be implemented in a data management system, but they have not yet been implemented. Thus, in this study we do not assess effects that are created by using our knowledge representation in a running medical information system, such as a biobank system.

The next step from the knowledge representation perspective is to develop computational ways to leverage the GCM system analysis and its results to immediately develop knowledge representation solutions such as OWL ontologies and RDF data. In addition, we plan to explore the deployment of the GCM to transform medical data captured with information models into computer-interpretable RDF data sources for secondary use in medicine and vice versa.

From the clinical perspective, the next step is to build a small proof-of-concept surgical biobank data management system following the knowledge representation principles demonstrated in this paper. This step, which we are currently working on, will allow validation of the functionality and usability of the proposed solutions in a clinical research setting.

## 9. Conclusions

From the results presented in this paper, we concluded that SWT is required to disambiguate and integrate data for surgical biobanks. While surgical biobanks with exclusively local data might not have the same level of need for semantic integration as those aiming to integrate data from multiple trauma care providers, we hold that integrating data from the entirety of the trauma care process is a necessity.

In addition, this paper demonstrates that the GCM is a useful tool to assess the data scope of a surgical biobank and identify relevant domain ontologies. The GCM even assists in resolving specific semantic integration problems, supplementing the SWTs.

## Figures and Tables

**Table 1:** Example answers to CAFÉ questionnaire.

CAFÉ Question	Trauma Center A (Level 2)	Trauma Center B (Level 1)	Trauma Center C (Level 1)
Number of emergency physicians who are board-certified in emergency medicine.	21	29	23
Number of emergency physicians who are board-eligible in emergency medicine	21	2	23

**Table 2:** Domains of orthopedic trauma care with examples of relevant entities and potential domain ontologies.

Domain	Types of Entities in Trauma Care	Potential Ontologies
**Resource Domain**	**Organization**: Trauma center, trauma system, emergency medical services (EMS), trauma team	OOSTT [10,11,12], OMRSE [34]
	**Human Individual**: Trauma patient, trauma medical director, trauma registrar, trauma surgeon, EMS personnel, plastic surgeon, infectiologist, microbiologist, surgical pathologist, endocrinologist, radiologist	
	**Facilities**: EMS vehicle, emergency room, trauma biobank, trauma registry	
**Policy Domain**	Resources for optimal care for injured patients; [35] EAST Practice Management Guidelines, [36] trauma system policies; triage plans; clinical guidelines	
**Medical Domain**	**Specimens**: Bone specimen; skin specimen; subcutaneous tissue specimen; small vein specimen, muscle specimen	OBIB [37], OBI [38], OMRSE [34], SNOMED-CT
	**Diagnosis**: Osteomyelitis, post-operative arthritis, pseudarthrosis,	
	**Treatment**: Resection, reconstruction, prosthesis, amputation, chronic injection therapy, nerve blocks for chronic pain	
	**Pathological process**: Inflammation, systemic inflammatory response syndrome, infection, necrosis, wound healing, bone regeneration, stress-induced hyperglycemia,	

## Data Availability

Not applicable.
